# Mitochondrial Dysfunction-Associated Arrhythmogenic Substrates in Diabetes Mellitus

**DOI:** 10.3389/fphys.2018.01670

**Published:** 2018-12-06

**Authors:** Jiajia Song, Ruilin Yang, Jing Yang, Lufang Zhou

**Affiliations:** ^1^Division of Cardiovascular Disease, Department of Medicine, University of Alabama at Birmingham, Birmingham, AL, United States; ^2^Key Laboratory of Mechanism Theory and Equipment Design of Ministry of Education, Tianjin University, Tianjin, China

**Keywords:** mitochondrial dysfunction, arrhythmogenesis, fibrosis, redox signaling, diabetes

## Abstract

There is increasing evidence that diabetic cardiomyopathy increases the risk of cardiac arrhythmia and sudden cardiac death. While the detailed mechanisms remain incompletely understood, the loss of mitochondrial function, which is often observed in the heart of patients with diabetes, has emerged as a key contributor to the arrhythmogenic substrates. In this mini review, the pathophysiology of mitochondrial dysfunction in diabetes mellitus is explored in detail, followed by descriptions of several mechanisms potentially linking mitochondria to arrhythmogenesis in the context of diabetic cardiomyopathy.

## Introduction

Diabetes mellitus (DM) is a group of chronic metabolic diseases that affects around 425 million people worldwide (Tuomilehto and Lindstrom, 2003). It is widely accepted that DM leads to a cascade of long-term severe complications, including cardiovascular diseases, renal failure and blindness (Association, 2000). Importantly, cardiovascular diseases account for nearly 70% of deaths in diabetic patients (Walker and Cubbon, 2015).

Diabetes mellitus can affect cardiac structure and function independently of vascular disease, hypertension or coronary artery disease, leading to the development of a heart disorder termed diabetic cardiomyopathy (DbCM). DbCM is characterized by a series of cardiac structural and functional remodeling, including left ventricular hypertrophy, interstitial fibrosis, lipid deposition, cell death, and decreased systolic dysfunction, which eventually lead to heart failure (Akar et al., 2005; Xie et al., 2009; Singh et al., 2018). In addition to contractile function, DbCM can also disrupt cardiac electrical activity, leading to arrhythmia (Jeong et al., 2016) and sudden cardiac death. It has been reported that patients with diabetes have a significantly increased risk of arrhythmogenesis compared with the general population (Huxley et al., 2011). Indeed, DbCM has become a major contributor to mortality in diabetic patients (Chen C. et al., 2018; D’Souza et al., 2018). Moreover, abnormal mitochondrial morphology (e.g., fragmented organelle, mottled matrix, and damaged membrane) and energetics have been observed in diabetes patients with arrhythmia (Montaigne et al., 2014; Montaigne and Pentiah, 2015), suggesting that mitochondrial dysfunction could contribute to arrhythmogenesis in the setting of DbCM.

In this mini view, we revisit some of the main features of DbCM, focusing on pathophysiological mechanisms associated with cardiac mitochondrial dysfunction, oxidative stress and fibrosis, and their proarrhythmic roles in DbCM.

## Mitochondria and Oxidative Stress in Diabetic Cardiomyopathy

**FIGURE 1:**
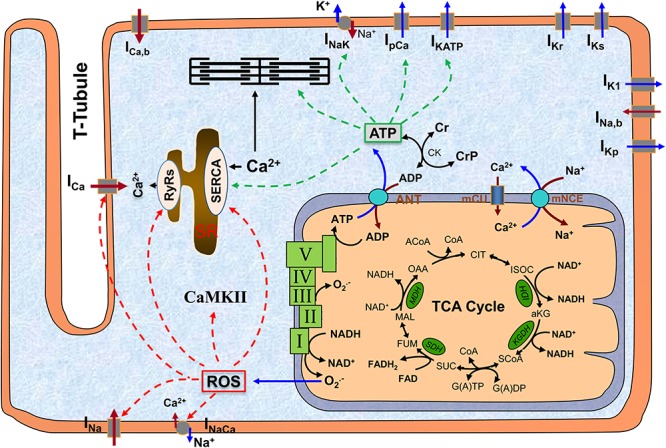
The scheme of mitochondrion and its interaction with other subcellular systems in cardiomyocyte. The major function of mitochondrion is to produce ATP, which occurs at complex V (*a.k.a.* F0F1 ATPase) using the electrochemical gradient generated by electron transport chain (complex I to IV). A byproduct of ATP production is superoxide (O_2_^.-^), which is probably generated at complex I and complex III. ATP is translocated to cytosol via ANT and hydrolyzed to support excitation-contraction and energy-sensitive ion transporters (indicated by green dashed lines). O_2_^.-^ can freely diffuse to cytosol and form ROS, which can modify a variety of redox sensitive ion transporters (indicated by red dashed lines). Altogether, mitochondrial dysfunction-associated ATP depletion and ROS accumulation can significantly affect cellular action potentials and ion homeostasis. ROS, reactive oxygen species; CaMKII, Ca^2+^/calmodulin-dependent protein kinase II; SR, sarcoplasmic reticulum; TCA, tricarboxylic acid; ANT, adenine nucleotide translocator; mCU, mitochondrial Ca^2+^ uniporter; mNCE, mitochondrial Na^+^/Ca^2+^ exchanger; I–V, complex I to complex V.

Mitochondria are the major powerhouse in cardiomyocytes, producing more than 95% of energy in the form of adenosine triphosphate (ATP). ATP is formed from adenosine diphosphate (ADP) and inorganic phosphate (Pi) by F_0_F_1_ ATP synthase (complex V), which uses the electrochemical gradient generated by the electron transport chain (complexes I to IV) driven by NADH produced in the tricarboxylic acid (TCA) cycle (Figure 1). ATP is transported into cytosol via adenine nucleotide translocator (ANT) and hydrolyzed to support cellular mechanical work (excitation and contraction), ion homeostasis, and molecular synthesis (Nicholls and Ferguson, 2002). Importantly, mitochondria are also a major source of reactive oxygen species (ROS) production (Chance et al., 1979), likely at complex I and complex III (Murphy, 2009; Figure 1). While physiological level ROS is essential for cellular redox signaling (Vanden Hoek et al., 1998), excessive ROS are toxic and can affect large molecules such as redox sensitive ion channels [e.g., ryanodine receptors (RyRs), sarcoplasmic reticulum (SR) Ca^2+^ transport ATPase (SERCA) and L-type Ca^2+^ channels (LCCs)]. Furthermore, mitochondria can directly regulate cytosolic Ca^2+^ via mitochondrial Ca^2+^ uniporter (mCU) and Na^+^/Ca^2+^ exchanger (mNCE). Not surprisingly, loss of mitochondrial function has been implicated to be closely associated with a variety of human diseases including DM (Bagheri et al., 2016; Jeong et al., 2016). In particular, studies have revealed that mitochondrial functional and structural alterations, including redox signaling, energy production, biogenesis, dynamics, and quality control, are important contributing factors to the pathogenesis of DbCM (Palikaras and Tavernarakis, 2014).

Oxidative stress is a hall marker of mitochondrial dysfunction, which is known to damage a variety of intracellular macromolecules, leading to lipid peroxidation and detrimental protein modifications (Zhang et al., 2012). Increased ROS levels may also activate multiple redox-sensitive signaling pathways involved in DbCM-relevant cardiac dysfunction, leading to impaired intracellular Ca^2+^ regulation, fibrosis, and loss of essential trace metal homeostasis. In the cardiomyocytes, the major site of ROS production is mitochondria (Chance et al., 1979; Murphy, 2009). In patients with diabetes, as glucose utilization is greatly impaired, the heart relies almost exclusively on fatty acids oxidation (FAO) to produce ATP. The switching to FAO leads to increased ROS production in DM (Block et al., 2009). Another important source of ROS production in DbCM is the hyperglycemia-induced advanced glycation end-products (AGEs) (Pal et al., 2014). In addition, it has been shown that inhibiting protein kinase C (PKC)-α signaling pathway reduces ROS levels and alleviates oxidative stress damage in DbCM in rats, suggesting that PKC-α is involved in ROS production in DM (Min et al., 2017).

## Mitochondrial-Associated Cardiac Arrhythmia

Cardiac arrhythmia refers to conditions in which the heart’s normal rhythm is disrupted, or the electrical activity is abnormal. Cardiac arrhythmias are traditionally considered to occur due to an abnormality in impulse initiation and/or electrical propagation. Abnormal impulse initiation is associated with triggered activity (i.e., trigger) resulting from premature activation of cardiac tissues by afterdepolarizations (e.g., early afterdepolarizations, EADs or delayed afterdepolarizations, DADs) or enhanced automaticity in the pacemaker cells, which may propagate as focal activity. Abnormal impulse propagation is associated with block of conduction or re-entry, which occurs when a single propagating impulse traveling through the heart gives rise to two or more propagated responses through wave-breaks. Reentrant arrhythmias can be attributed to three main cellular mechanisms: (i) loss of cell to cell electrical coupling and communication by closure of gap junctions, (ii) regional uncoupling caused by anatomical barriers such as scar tissue, and (iii) dynamic functional block due to heterogeneity of intrinsic electrophysiological restitution properties. The detailed review of mechanism of cardiac arrhythmogenesis can be found here (Antzelevitch and Burashnikov, 2011; Tse, 2016).

While arrhythmia is a leading cause of sudden cardiac death in patients with diabetes (Cohn et al., 1986; Cohn, 1996), the precise molecular mechanisms underlying cardiac arrhythmogenesis in DbCM are poorly understood, hindering the development of effective therapeutic strategy. Recently, the loss of mitochondrial function, which is often observed in DbCM, has emerged as a key contributor to the arrhythmogenic substrates (Li et al., 2008; Xie et al., 2013), probably via modulating the redox and/or energy sensitive signaling pathways that regulate ion handling channels/transporters (Maack and O’Rourke, 2007; Yan et al., 2008; Zhou et al., 2009, 2011).

### Oxidative Stress-Associated Arrhythmic Substrates

Although hyperglycaemia regulates multiple pathways associated with the pathogenesis of DbCM, oxidative stress is considered as a central mechanism underlying the adverse remodeling in diabetic hearts (Liu Q. et al., 2014), including electrophysiological alternations (Li et al., 2008; Karam et al., 2017). Both *in vitro* and computational studies have shown that ROS cause action potential duration (APD) prolongation and induce EADs or DADs in guinea pig (Liu T. et al., 2014; Li et al., 2015) and rabbit cardiomyocytes (Xie et al., 2009), leading to focal activity. ROS can also promote reentry via heterogeneous APD prolongation (Morita et al., 2011). It is worth noting that both reentry and focal activity account for ROS-mediated arrhythmogenesis.

At the cellular level, the proarrhythmic effect of ROS is attributed to their capability to modulate multiple redox ion channels/transporters underlying ion handling and action potentials (Howe et al., 2004; Zima and Blatter, 2006; Wagner et al., 2013), which include RyRs (Eager et al., 1997; Gen et al., 2001; Yan et al., 2008; Zhou et al., 2011), SERCA (Morris and Sulakhe, 1997; Zima and Blatter, 2006), voltage-gated Na^+^ channels (Na_v_) (Liu et al., 2010; Jeong et al., 2012), K^+^ channels (K_ir_ and K_v_) (Zhang et al., 2006), Na^+^/Ca^2+^ exchanger (NCX) (Bers and Despa, 2006), and LCCs (Barrington et al., 1988; Coetzee and Opie, 1992; Nakaya et al., 1992; Ward and Giles, 1997; Kourie, 1998; Zima and Blatter, 2006). One of the most well-characterized redox-sensitive ion transporters is RyRs, which have 89 cysteine residues, of which approximately 21 are susceptible to oxidation by free radicals (Xu et al., 1998). Studies have shown that extracellular H_2_O_2_ activates RyRs, leading to increased SR Ca^2+^ release in rat (Gen et al., 2001; Oda et al., 2015) and sheep (Eager et al., 1997) ventricular myocytes. Moreover, recent studies have shown that mitochondrial-derived ROS (mdROS) are closely correlated with enhanced Ca^2+^ sparks in resting guinea pig cardiomyocytes (Yan et al., 2008; Zhou et al., 2011). In addition to RyRs, ROS can affect SR Ca^2+^ uptake. It has been shown that ROS inhibit SERCA activity by directly oxidizing its thiol groups (Morris and Sulakhe, 1997; Zima and Blatter, 2006). Thus, ROS, by simultaneously activating RyRs and inhibiting SERCA, may lead to increased Ca^2+^ transients (Ward and Moffat, 1995; Li et al., 2015). With regards to diabetes, studies have found that free fatty acids-induced mdROS contributes to Ca^2+^ dysregulation, likely by triggering aberrant endoplasmic reticulum/SR Ca^2+^ release, leading to increased DADs and probability of focal excitation (Roussel et al., 2015; Ly et al., 2017).

Sarcoplasmic Na^+^ channels encoded by the SCN5A gene are key to cardiac excitability and rapid impulse propagation. Song et al. (2006) have demonstrated that the late Na^+^ current is involved in H_2_O_2_-induced APD prolongation, EADs and DADs in both guinea pig and rabbit cardiomyocytes. Another study showed that ranolazine, a late Na^+^ current blocker, suppressed ROS-mediated EAD and arrhythmias (Morita et al., 2011). Importantly, while ROS-induced increase in late Na^+^ current can elicit EADs and arrhythmia, increased ROS may downregulate the total Na^+^ channel expression, resulting in reduced Na^+^ currents and conduction velocity, providing substrates for reentry (Liu et al., 2010).

In addition to directly modulating redox sensitive ion channels/transporters, ROS can also indirectly influence ion homeostasis and consequently action potentials via redox signaling such as Ca^2+^/calmodulin dependent kinase II (CaMKII) oxidation. CaMKII is a ubiquitously expressed multifunctional protein kinase that can be activated by binding to Ca^2+^/calmodulin (Zhang and Brown, 2004; Wagner et al., 2013). Recent studies suggest that CaMKII can also be activated by ROS (Erickson et al., 2008, 2011; Huke and Knollmann, 2011; Joiner et al., 2012), leading to phosphorylation of a wide range of ion handling proteins such as Na^+^ channels (Wagner et al., 2006, 2011), LCCs (Dzhura et al., 2000; Hudmon et al., 2005; Grueter et al., 2006; Blaich et al., 2010), RyRs (Maier et al., 2003; Wehrens et al., 2004; Guo et al., 2006; Kohlhaas et al., 2006; Maier and Bers, 2007; Sag et al., 2013; Ho et al., 2014), and phospholamban (Bassani et al., 1995; Maier and Bers, 2007; Sag et al., 2013). Xie et al. (2009) have showed that H_2_O_2_ perfusion-induced oxidative CaMKII activation leads to afterdepolarizations in isolated rabbit cardiomyocytes, likely via phosphorylation of Na^+^ channels and LCCs. In a computational study, Yang R. et al. (2018) reported that mdROS-mediated oxidative CaMKII activation induces EADs in guinea pig cardiomyocytes by enhancing the late component of Na^+^ current. The proarrhythmic role of CaMKII oxidation in diabetic hearts has been demonstrated in a recent study showing that selective genetic blocking of CaMKII oxidation prevents the enhanced atrial fibrillation (AF) risk (Mesubi et al., 2017), which has been implicated to be associated with increased mortality in response to myocardial infraction (Luo et al., 2013). Redox signaling can also regulate several pro-arrhythmic transcription factors such as nuclear factor-κB (NF-κB), one of the key transcriptional regulators that mediates gene expressions under stress conditions including diabetes. Importantly, Dudley and colleagues have shown that the promoter region of the SCN5A gene encoding cardiac Na^+^ channels contains a NF-κB binding domain, suggesting that SCN5A can be regulated by NF-κB in response to oxidative stress (Shang et al., 2008).

### Energy Deficiency-Associated Arrhythmias

Studies have shown that oxygen consumption rate and ATP synthesis are reduced in patients with insulin resistance (Szendroedi et al., 2012) and animals fed with high palmitate (Xu et al., 2015), which can contribute to the development of arrhythmia. A series of studies by O’Rourke’s laboratory have convincingly demonstrated that mitochondrial depolarization-induced ATP depletion elicits reentrant arrhythmias via a mechanism termed “metabolic sink” (Akar et al., 2005; Brown et al., 2008; Aon et al., 2009; Zhou et al., 2014). In particular, they showed that decreased ATP levels, or increased ADP/ATP ratio, caused by mitochondrial depolarization, leads to rapid activation of the sarcoplasmic potassium-sensitive K_ATP_ (K_ATP_) channels, causing shortening of APD and reduction of action potential amplitude (APA) (Zhou et al., 2009). When a threshold of opening K_ATP_ channels is reached, cardiomyocytes are rendered completely inexcitable. They further showed that mitochondrial oscillations [a phenomena involving local oxidative stress-induced cyclic changes in mitochondrial membrane potential, NADH oxidation and ROS production (Aon et al., 2003; Zhou and O’Rourke, 2012; Zhou et al., 2014)] cause highly correlated, repetitive activations of the K_ATP_ channels and fluctuations in both APD and APA (Zhou et al., 2009), indicating that there is a direct link between mitochondrial energetic dysfunction and arrhythmia. At the tissue level, regional mitochondrial depolarization and K_ATP_ current activation can create inhomogeneous regions or “metabolic sink,” in which the increased dispersion of repolarization enhances the vulnerability to reentry, as revealed by both experimental studies (Akar et al., 2005) and computer simulations (Zhou et al., 2014).

Other ATP-sensitive ion transporter proteins include SERCA and sarcoplasmic Na^+^/K^+^ ATPase. It has been shown that expression of ATP2A2 gene that encodes SERCA2a, an isoform mainly expressed in cardiomyocytes, is reduced in DbCM (Suarez et al., 2008). Studies also showed that reduced mitochondrial ATP production suppresses SERCA activity (Landolfi et al., 1998; De Marchi et al., 2011). The decreased SERCA2a activity and expression present in the diabetic myocardium has been considered as a main contributing factor to impaired Ca^2+^ cycling and aberrant action potentials. Similarly, ATP depletion can decrease Na^+^/K^+^ ATPase activity (Ziegelhoffer et al., 2000), an electrogenic pump on sarcoplasmic membrane, leading to not only dysregulation of ion homeostasis but also alteration of the membrane potential.

In addition to influence cellular ion channels and action potentials, decreased ATP production can alter intercellular coupling via gap junctions. Gap junctions are hemichannels in the heart that form electrical connection between cells, mediating the spread of electrical impulse and coordinated contraction of cardiac chambers (see review Sohl and Willecke, 2004). Connexin 43 (Cx43) is the major component of gap junctions in the ventricular myocytes. In heart failure, the level of Cx43 expression is reduced, which is associated with depressed AP propagation and increased incidence of lethal ventricular arrhythmias. At the single channel level, studies have shown that Cx43 conductance can be inhibited by decreased intracellular ATP concentration in guinea pig (Sugiura et al., 1990) and rat (Duthe et al., 2001) cardiomyocytes, among others (see review Schulz and Heusch, 2004), causing suppression of intercellular electrical conductance. It is worth mentioning that gap junction conductance can also be impaired by oxidative stress (Berthoud and Beyer, 2009; Jeong et al., 2012).

## Mitochondrial Dysfunction, Fibrosis and Atrial Fibrillation in Diabetes

Cardiac fibrosis is characterized by fibroblasts accumulation and excessive deposition of extracellular matrix (ECM) that greatly increases the stiffness of the heart wall and reduces the contractility and compliance of cardiac muscle (Fan et al., 2012). Myocardial fibrosis is increasingly recognized as one of the major factors contributing to the pathogenesis of DbCM (Shen et al., 2014). In addition, fibrosis, particularly atrial interstitial fibrosis, increases vulnerability to arrhythmia because of delayed discontinuous and zig-zag conduction and unidirectional conduction block (De Jong et al., 2011; Nguyen et al., 2014).

### Mitochondrial Dysfunction and Pathogenesis of Cardiac Fibrosis in DbCM

In the heart, fibrosis can be activated by multiple sources, including increased mechanical stretch (Froese et al., 2016), dysregulated redox signaling (Dai et al., 2011), paracrine cytokines from injured cells (Yue et al., 2013), and infiltration of circulatory immune cells (Ismahil et al., 2014). Among those, the transforming growth factor β1 (TGF-β1) is recognized as a major fibrogenic factor. In diabetes, hyperglycemia changes the levels of microRNAs and long non-coding RNAs expression, which can contribute to TGF-β activation (Yue et al., 2013). TGF-β activity can also be increased by high glucose-induced activation of transcriptional co-regulator p300 (Bugyei-Twum et al., 2014), sustained hyperglycemia (Wu et al., 2016; Xu et al., 2016), and activation of matrix metalloproteinases (MMP), a family of ECM proteolytic enzymes (Dayer and Stamenkovic, 2015; Li et al., 2018). Activated TGF-β1 binds to its membrane receptors phosphorylates transcription factors SMAD2/3, which activates the canonical (SMAD-dependent) fibrogenic pathway (Verrecchia and Mauviel, 2002; Khalil et al., 2017), leading to myofibroblast differentiation and fibrosis (Lijnen et al., 2003). TGF-β1 can also stimulate endothelial-to-mesenchymal transition (EndMT) through convergence of SMAD-dependent and SMAD-independent signaling (Medici et al., 2011). Activated EndMT can increase the expression levels of mesenchymal markers, such as α-smooth muscle actin (α-SMA), fibroblast-specific protein-1, and vimentin (Medici and Kalluri, 2012), promoting cardiac fibrosis in diabetic hearts. Importantly, studies have implicated an interplay between TGF-β1 signaling and mitochondrial dysfunction and associated redox signaling (Cucoranu et al., 2005). On one hand, TGF-β1 can upregulate NADPH oxidase 4 (NOX4) expression, resulting in increased mitochondrial ROS production (Jiang et al., 2014). On the other hand, mdROS have been shown to activate TGF-β1 signaling (Koitabashi et al., 2011). Thus, there is a positive feedback mechanism between TGF-β1 and mitochondrial-associated ROS production, facilitating the amplification of the profibrogenic TGF- β1 signaling in DbCM (Dudley et al., 2005; Anderson et al., 2009).

In addition to TGF-β1, some members of the mitogen-activated protein kinase (MAPK) family and protein kinase B (Akt)/glycogen synthase kinase-3β (GSK-3β) signaling, are also involved in myocardial fibrosis (Ying et al., 2017; Yue et al., 2017). Suppression of the MAPKs signaling or the Akt/GSK-β signaling has been shown to ameliorate myocardial fibrosis in diabetes (Liu et al., 2016; Zhang et al., 2016). The PKC, which is activated under oxidative stress, can also contribute to myocardial fibrosis by activating NF-κB and the sequential collagen accumulation (Bhattacharjee et al., 2017). The inhibition of redox sensitive NF-κB signaling has been shown to attenuate hyperglycemia-induced cardiac injuries (Chen X. et al., 2018).

In summary, mdROS, which is increased in diabetic human atrial tissue (Anderson et al., 2009), could play important roles in the pathophysiology of cardiac fibrosis in DbCM, among many other contributing factors, likely through regulating various redox signaling pathways.

### Cardiac Fibrosis and Atrial Fibrillation in DbCM

Atrial fibrillation (AF) is characterized by rapid and uncoordinated atrial activity that causes ineffective atrial contraction. As the most common sustained arrhythmia in clinical practice, AF causes major cardiovascular morbidity and mortality in DM patients (Karam et al., 2017). A recent cross sectional survey in China reported that the prevalence of AF in patients with DM is significantly higher than those without known DM (Hu and Sun, 2008). Another study in Japan showed the similar findings that the incidence of AF in diabetic patients is much higher than in the control group (Iguchi et al., 2008).

Recently, DbCM-related excessive fibrosis has emerged as a hallmark and independent risk factor for both triggering and sustaining AF (Movahed et al., 2005). Myocardial fibrosis in the atrial tissue can elicit AF by causing abnormal cell coupling, which has been observed in DM patients. Cardiac fibroblasts (CF) are non-excitable cells that obstruct the orderly spread of electrical impulse in the hearts by producing an insulating layer of ECM to physically separate groups of myocytes. Thus, excessive CF forms collagen-rich myocardial tissue that causes reduced inter-cardiomyocyte coupling and increased axial resistance, leading to impaired propagation of cardiac action potentials and formation of reentrant excitations. On the other hand, CF can couple to myocytes via gap junction proteins Cx43 and connexin-45 (Kohl, 2003; Chilton et al., 2007) and studies have shown that the level of Cx43 expression increases significantly in diabetic rat myocardium (Watanabe et al., 2012). As CF have a high cell membrane resistance that makes them excellent long-distance conductors (Kohl, 2003), increased coupling between CF and cardiomyocytes will decrease the conduction velocity and induce synchronous spontaneous activity in distant cardiomyocytes (Viennet et al., 2003; Abdullah et al., 2016). In addition, studies have revealed that atrial effective refractory period (AERP) is shortened in the diabetic rat atrium, which results in increased dispersion of AERP and formation of unidirectional conduction block (Otake et al., 2009), predisposing to reentrant AF.

### Mitochondrial-Derived Oxidative Stress and Atrial Electrical Remodeling in DbCM

In addition to promoting arrhythmogenesis through enhancing fibrosis and structural remodeling, mdROS are also involved in the proarrhythmic electrical remodeling in DM [readers are referred to Koektuerk et al. (2016) and Karam et al. (2017) for comprehensive reviews]. For instance, a recent study has shown that dipeptidyl peptidase-4 inhibitor prevents mitochondrial dysfunction (e.g., mdROS production and mitochondrial depolarization) and ameliorates atrial remodeling and arrhythmic substrates in the diabetic rabbits (Zhang et al., 2017). The potential link between mdROS and atrial remodeling in the setting of DM has also been suggested by another study showing that allopurinol, a xanthine oxidase inhibitor, prevents alloxan-induced atrial interstitial fibrosis, abnormal Ca^2+^ handling and atrial fibrillation inducibility in rabbits (Yang et al., 2018). Furthermore, studies have revealed significant alterations of electrical structure such as effective refractory period (Otake et al., 2009) and gap junction conduction (Joshi et al., 2015) in the atrial of diabetic animals. These studies suggest that mitochondrial dysfunction-induced atrial remodeling may underly the onset and maintaining to AF.

## Summary

**FIGURE 2:**
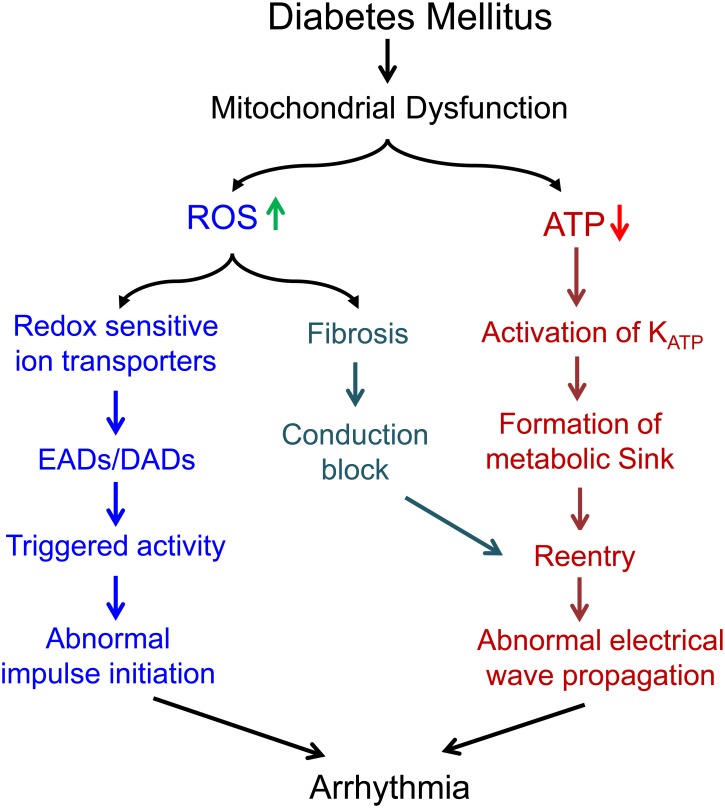
Summary of the potential mechanisms underlying the proarrhythmic role of mitochondrial dysfunction in diabetes mellitus.

Mitochondrial dysfunction plays a potential role in the generation and maintenance of cardiac arrhythmias in diabetic hearts. The loss of function of mitochondria is associated with not only reduced ATP production, increased ROS production and acute changes in ion levels, but also dysregulation of multiple signaling pathways regulating transcript expression and fibrosis. All of those could contribute to cardiac arrhythmogenesis (Figure 2), suggesting that mitochondria could be an upstream therapeutic target for treatment of arrhythmias in DbCM.

## Author Contributions

All authors prepared the manuscript. LZ finalized the manuscript.

## Conflict of Interest Statement

The authors declare that the research was conducted in the absence of any commercial or financial relationships that could be construed as a potential conflict of interest.
